# Combined Impact of Traditional and Non-Traditional Healthy Behaviors on Health-Related Quality of Life: A Prospective Study in Older Adults

**DOI:** 10.1371/journal.pone.0170513

**Published:** 2017-01-25

**Authors:** Ana Bayán-Bravo, Raúl F Pérez-Tasigchana, Carmen Sayón-Orea, David Martínez-Gómez, Esther López-García, Fernando Rodríguez-Artalejo, Pilar Guallar-Castillón

**Affiliations:** 1 Department of Preventive Medicine and Public Health, School of Medicine, Universidad Autónoma de Madrid /IdiPaz, CIBER of Epidemiology and Public Health (CIBERESP), Madrid, Spain; 2 Nefrology Department, "12 de Octubre" Hospital, Madrid, Spain; 3 School of Medicine, Universidad San Francisco de Quito, Quito, Ecuador; 4 Complejo Hospitalario de Navarra, Pamplona, Spain; 5 Department of Physical Education, Sports and Human Movement, Universidad Autónoma de Madrid, Madrid, Spain; Texas Technical University Health Sciences Center, UNITED STATES

## Abstract

Combined exposure to several healthy behaviors (HB) is associated with reduced mortality in older adults but its impact on health-related quality of life (HRQL) is uncertain. This is a cohort study of 2,388 individuals aged ≥60 recruited in 2000–2001, whose data were updated in 2003 and 2009. At baseline, participants reported both traditional HB (non-smoking, being very or moderately active, healthy diet) and non-traditional HB (sleeping 7–8 h/d, being seated <8 h/d, and seeing friends every day). HRQL was measured with the SF-36 questionnaire at baseline, in 2003 (short-term) and in 2009 (long-term); a higher score on the SF-36 represents better HRQL. Linear regression models were used to assess the association between HB at baseline and HRQL in 2003 and 2009, with adjustment for the main confounders including baseline HRQL. In the short-term, being physically active, sleeping 7–8 h/d, and being seated <8 h/d was associated with better HRQL. Compared to having ≤1 of these HB, the β (95% confidence interval) for the score on the physical component summary of the SF-36 in 2003 was 1.42 (0.52–2.33) for 2 HB, and 2.06 (1.09–3.03) for 3 HB, p-trend <0.001. Corresponding figures for the mental component summary score were 1.89 (0.58–3.21) for 2 HB and 3.35 (1.95–4.76) for 3 HB, p-trend <0.001. Non-smoking, a healthy diet or seeing friends did not show an association with HRQL. In the long-term, being physically active was the only HB associated with better physical HRQL. As a conclusion, a greater number of HB, particularly more physical activity, adequate sleep duration, and sitting less, were associated with better short-term HRQL in older adults. However, in the long-term, being physically active was the only HB associated with better physical HRQL.

## Introduction

There is evidence of the benefits of a healthy lifestyle in old age. Specifically, several healthy behaviors (HB), including traditional factors (non-smoking, physical activity, and healthy diet) and non-traditional factors (adequate sleep duration, avoiding sedentariness, and a good social network) have been associated with reduced mortality in older adults; moreover, there is an inverse dose-response between the number of HB and mortality.[1]This approach, that considers the combined exposure to several HB, is relevant because it captures their synergistic impact; in fact, HB do not occur in isolation, but they aggregate in the population.[2–4]

A complete assessment of the impact of HB should also include health outcomes other than mortality. This is particularly important in older adults, whose life expectancy is limited; in fact, in this population subgroup, adding health to years is a key personal and social objective.[5] In the last decades, life expectancy has increased substantially in many countries, but heath status, including physical function, is still poor among the elderly.[5] Many health problems in older people result from chronic diseases that could be prevented or palliated by appropriate HB. Thus, healthy ageing through the adoption or maintenance of HB among older people is still a challenge to be addressed in the future.

Health-related quality of life (HRQL) represents how an individual perceives their own health status, and provides subjective information on physical, social and mental health aspects.[6] As a result, a poor HRQL predicts higher mortality[7] and greater use of healthcare services, including hospital admission.[8] A number of recent studies have assessed the combined effect of several HB on HRQL,[9,10] and have found that the accumulation of unhealthy behaviors is associated with poor HRQL. Specifically, the "Midlife in the United States" study (MIDUS) examined the joint protective contribution of control beliefs, social support and physical exercise to changes in functional health, and found that age-related declines in health were reduced among those with more protective factors, fostering the maintenance of functional health.[9] Also, a cross-sectional study in Australia investigated the combined influence of smoking, alcohol consumption, physical activity, diet, sitting time, and sleep duration on HRQL, and found that engaging in a greater number of poor lifestyle behaviours was associated with a higher prevalence of poor HRQL.[10]

However, these studies were conducted mostly with middle-aged individuals. As such and to our knowledge, this is the first study to assess the association between a healthy lifestyle and HRQL in older adults; specifically, it examined the short- and long-term combined impact of traditional and non-traditional HB on HRQL in older adults from Spain.

## Materials and Methods

Informed consent was given by each participant in the study. The research protocol was approved by the Clinical Research Ethics Committee of "La Paz" University Hospital in Madrid, Spain. All the participants gave written consent.

The methods of this study have been reported elsewhere.[11,12] Briefly, data were taken from a prospective cohort of 4,008 individuals (1,739 men and 2,269 women) aged 60 and older who were recruited in 2000–2001 in Spain. The study participants were selected by probabilistic and multistage cluster sampling. The clusters were stratified by region of residence and size of municipality. A total of 420 census tracts were randomly selected within clusters, and participants were chosen according to age and sex strata in each tract. Individuals who did not participate after 10 visits, those who had died or were institutionalized at the moment of data collection, and those who refused to participate were replaced with the same sampling procedure. In the analyses, each subject was weighed according to their sex, age, region of residence and size of the municipality, to reflect the characteristics of the population of older Spanish adults. Data were collected at home with an interview and physical examination performed by trained and certified staff. The study response rate was 71%.

In 2003, the study participants were contacted again. After excluding 245 who had died, we obtained information on 2,388 individuals through a telephone interview. Those contacted did not differ significantly from those lost to follow-up in socio-demographic characteristics and lifestyle, except for the number of diagnosed chronic conditions in 2001, that was 1.2 among those who remain in the cohort, and 1.4 among those lost during follow-up.[13]

Lastly, in 2009 another wave of data collection was performed. After excluding 1,105 who had died since 2003, telephone interviews were conducted with 1,323 individuals. Compared with those who were lost to follow-up between 2003 and 2009, those who continued in the study were younger, had a higher level of education, were less sedentary, and had a fewer number of chronic diseases.[14]

Traditional and non-traditional HB were chosen according to public health guidelines, [15,16] systematic reviews of the literature,[17,18] and findings of a recent analysis in this cohort.[1] As regards traditional HB, individuals reported their smoking status (never, former or current smoker); former smokers also indicated the time since smoking cessation, and never smoking or having quit at least 15 years ago was considered a HB. Physical activity (PA) was assessed with a single global question that classified subjects as very active, moderately active, less active or inactive, as compared with people of their age.[19] Being very or moderately active were deemed to be a HB. Food consumption was ascertained with a simplified 14-item instrument developed from a validated food-frequency questionnaire.[20,21] Consumption of fruit, vegetables, whole grains, red or processed meat, animal and vegetable fat, and fish was used to build a healthy diet index. For each group of foods, the following consumption categories were considered: every day, 3–5 days a week, 1–2 days a week, or never. We assigned +1 point to each of the following categories: fruit every day, vegetables every day, whole grains every day, vegetable oils every day, and fish at least three days a week; we also assigned -1 point for consuming red meat or processed meat every day, or animal fats every day. A lower score indicates a worse diet; we defined a healthy diet as a score ≥ the median in the cohort.

Regarding non-traditional HB, sleep duration was obtained with the question: How many hours do you usually sleep per day (including both night-time and daytime sleep)?[22] Participants had a HB if they slept 7 to 8 hours/day. Sedentary lifestyle was estimated by leisure time spent sitting down, based on the following question: How much time do you spend sitting during weekdays? Please indicate the total hours of all these activities (eating, listening to the radio, watching TV, reading, sewing, driving, etc.).[12] The same question was asked about the weekends. The number of hours spent seated per day were calculated as follows: [(time sitting on weekdays X 5 + time sitting on weekends X 2)/7)]. Being seated <8 hours/day was considered as a HB. Finally, the social network was represented by the frequency (every day, 1–2 times a week, 1–2 times a month, every few months, rarely or never) with the individuals saw their friends or neighbours.[23] Seeing them every day was deemed as healthy.

HRQL was measured at baseline, in 2003, and 2009 using the SF-36 questionnaire.[24] This instrument consists of 36 items, that measure the following eight dimensions of HRQL: physical functioning, physical role, bodily pain, general health, vitality, social functioning, emotional role, and mental health. Physical functioning, physical role, and body pain reflect mainly the physical health dimension; social functioning, emotional role and mental health mainly represent psychosocial and mental health; and vitality and general health are associated with both physical and mental HRQL. The subjects' responses to each of the items were scaled from 0 to 100, with a higher score representing better HRQL. In general, differences of 3–5 points in each scale are considered clinically relevant.[25] The SF-36 also allows to build a physical component summary (PCS) and a mental component summary (MCS), where higher scores indicate better HRQL. The Spanish version of the SF-36 has been previously used to measure HRQL in the elderly, and has demonstrated good reproducibility and validity. [24,26,27]

We recorded age, sex, education (no formal, primary, secondary or higher education) and employment status (employed, unemployed, retired, and housewives). Consumption of beer, wine and spirits was assessed with a quantity-frequency questionnaire, that served to classify individuals as non-drinkers, former-, moderate- or excessive-drinkers.[28] To minimize recall bias in sleeping disorders, analyses were adjusted for extreme sleep durations (≤3 or ≥16 h/d).

Weight, height and waist circumference (WC) were measured using standardized procedures.[29] Body mass index (BMI) was calculated as weight (kg) divided by squared height (m^2^). General obesity was defined as BMI ≥30 kg/m^2^, and abdominal obesity as WC >102 cm in men and >88 cm in women. Blood pressure was measured six times in the right arm at the heart level with standardized methods.[30] The average of the six measurements was used in the analyses. Hypertension was defined as systolic blood pressure ≥140 mmHg. Participants were considered as hypercholesterolemic when they answered positively to this question: Did your physician ever tell you that you had high blood cholesterol?

Finally, the following self-reported physician-diagnosed diseases were recorded: coronary heart disease, stroke, diabetes mellitus, hip fracture, cancer at any site, chronic obstructive pulmonary disease, and osteoarthritis.

Among 2,388 individuals who provided updated information in 2003, 295 were excluded because of missing data on HB, HRQL and potential confounders. Thus, the analyses using data at baseline and in 2003 were performed with 2093 individuals. Among the 1,323 participants in 2009, 330 were excluded due to missing data on study variables. Therefore, analyses in 2009 were conducted with 993 individuals.

Baseline characteristics of the participants were age-adjusted using the Willett's residual method.[31] Linear regression models were fitted using the SF-36 scales and summaries as dependent variables, and the traditional and non-traditional HB as the main independent variables. The models were adjusted for the potential confounders described above and for HRQL at baseline. To calculate the p for linear trend, the number for HB was introduced as a continuous variable. Analyses were performed with Stata/MP v13.

## Results

At baseline, men scored higher than women in all SF-36 scales and summaries. Higher education and higher alcohol intake were also associated with better HRQL. By contrast, individuals with extreme sleep duration, general or abdominal obesity, and the rest of morbidities had lower scores on HRQL (Table 1).

**Table 1:** Age-adjusted mean (standard deviation) of the SF-36 scales in 2000–2001, according to characteristics of study participants. (n = 2,093).

	Physical functioningMean (SD)	Physical roleMean (SD)	BodilypainMean (SD)	General healthMean (SD)	VitalityMean (SD)	Social functioningMean (SD)	Emotional roleMean (SD)	Mental healthMean (SD)	Physical SummaryMean (SD)	Mental SummaryMean (SD)
**Total**	72.6 (24.0)	78.3 (37.4)	70.8 (28.9)	58.5 (20.6)	64.0 (23.7)	83.1 (25.1)	87.4 (30.0)	70.0 (22.0)	45.5 (9.8)	49.8 (11.0)
**Socio-demographic variables**										
**Men**	78.8 (21.3)	84.8 (32.3)	78.2 (25.7)	62.7 (20.3)	71.1 (21.4)	89.4 (20.2)	93.1 (22.6)	77.6 (19.2)	47.5 (8.6)	53.0 (8.7)
**Women**	68.0 (24.9)	73.4 (40.1)	65.3 (29.9)	55.4 (20.2)	58.7 (24.1)	78.4 (27.3)	83.1 (33.8)	64.4 (22.3)	44.0 (10.4)	47.4 (11.9)
**Educational level**										
**No formal education**	68.0 (25.1)	74.6 (39.7)	67.6 (30.4)	53.4 (19.0)	60.5 (23.8)	79.1 (27.3)	85.0 (32.7)	67.0 (22.0)	43.7 (10.1)	48.6 (11.5)
**Primary education**	75.9 (22.3)	81.6 (34.6)	72.3 (27.6)	62.0 (21.0)	66.3 (23.9)	85.6 (23.7)	88.8 (28.0)	71.5 (22.7)	46.9 (9.3)	50.3 (10.9)
**Secondary education or higher**	81.9 (19.4)	86.3 (30.9)	80.0 (24.4)	67.6 (19.4)	73.2 (19.5)	90.5 (18.3)	93.9 (20.5)	78.8 (17.8)	48.9 (8.0)	53.2 (8.3)
**Occupational status**										
**Employed**	72.5 (23.1)	81.7 (33.4)	72.5 (29.0)	60.5 (20.4)	64.1 (22.3)	84.4 (25.6)	89.7 (27.3)	72.3 (20.4)	45.9 (9.7)	50.7 (10.5)
**Unemployed**	54.3 (31.0)	89.0 (26.6)	62.5 (34.5)	50.9 (15.6)	51.3 (27.0)	72.3 (34.9)	95.7 (20.6)	74.4 (17.5)	39.9 (11.5)	51.6 (9.1)
**Retired**	72.8 (24.0)	77.9 (37.8)	70.7 (28.8)	58.5 (20.6)	64.3 (23.9)	83.3 (24.8)	87.3 (30.0)	70.1 (22.1)	45.5 (9.8)	49.9 (11.0)
**Housewives**	72.1 (23.4)	73.6 (39.9)	69.3 (30.7)	52.3 (19.0)	56.3 (22.3)	75.6 (29.3)	77.4 (38.4)	58.5 (22.3)	45.9 (10.3)	43.8 (13.2)
**Lifestyles**										
**Alcohol intake**										
**Non-drinker**	68.4 (24.7)	73.2 (40.2)	67.0 (29.8)	53.8 (19.3)	58.9 (23.4)	78.1 (27.0)	85.4 (31.5)	65.7 (22.4)	43.7 (10.0)	58.9 (23.4)
**Ex-drinker**	66.4 (24.3)	77.1 (36.8)	65.1 (30.9)	52.5 (20.7)	61.1 (25.1)	79.8 (28.3)	84.1 (32.2)	66.4 (24.0)	43.5 (10.2)	61.1 (25.1)
**Moderate consumption**	78.9 (22.1)	83.7 (33.8)	75.7 (26.6)	64.8 (20.6)	69.3 (22.9)	88.5 (21.6)	90.0 (28.3)	74.5 (20.5)	48.0 (9.2)	69.3 (22.9)
**Excessive consumption**	78.4 (20.7)	85.3 (31.7)	78.2 (26.0)	65.8 (18.6)	72.9 (20.5)	91.9 (16.8)	91.4 (24.8)	78.9 (17.0)	47.9 (8.1)	72.9 (20.5)
**Extreme sleep durations**										
**No**	72.7 (24.0)	78.4 (37.3)	70.9 (28.9)	58.6 (20.6)	64.1 (23.7)	83.3 (25.1)	87.6 (29.7)	70.3 (21.8)	45.5 (9.8)	49.9 (11.0)
**Yes**	69.3 (25.5)	72.5 (42.4)	66.4 (30.6)	52.9 (19.5)	57.9 (26.6)	76.4 (28.1)	77.1 (40.0)	54.3 (26.6)	45.6 (9.2)	43.4 (12.7)
**Body Mass Index**										
**<30 Kg/m**^**2**^	76.0 (22.4)	81.2 (35.7)	73.9 (27.3)	60.3 (19.9)	66.0 (22.8)	85.9 (22.8)	88.8 (28.6)	71.6 (20.9)	46.8 (9.3)	50.3 (10.4)
**≥30 Kg/m**^**2**^	67.2 (25.5)	73.5 (39.5)	65.9 (30.7)	55.6 (21.3)	60.8 (24.9)	78.7 (28.0)	85.1 (32.0)	67.6 (23.5)	43.5 (10.2)	48.9 (11.8)
**Waist circumference**										
**No abdominal obesity**	78.4 (21.0)	83.1 (33.9)	75.3 (26.5)	61.1 (20.4)	69.3 (22.2)	86.9 (21.9)	90.7 (25.4)	73.2 (20.9)	47.4 (8.7)	51.2 (9.8)
**Abdominal obesity**^a^	69.8 (24.9)	75.9 (38.8)	68.6 (29.8)	57.3 (20.5)	61.5 (24.1)	81.3 (26.4)	85.8 (31.8)	68.5 (22.4)	44.6 (10.2)	49.1 (11.5)
**Systolic blood pressure**										
**<140 mmHg**	72.6 (24.0)	79.4 (36.8)	70.8 (28.9)	58.9 (20.5)	64.2 (23.6)	83.1 (24.9)	87.0 (30.7)	70.4 (22.1)	45.7 (9.8)	49.8 (11.1)
**≥140 mmHg**	72.7 (24.0)	77.3 (37.9)	70.9 (28.9)	58.2 (20.6)	63.9 (23.9)	83.1 (25.3)	87.8 (29.3)	69.7 (21.9)	45.4 (9.8)	49.8 (10.9)
**Hypercholesterolemia**										
**No**	73.3 (23.8)	78.9 (37.2)	72.1 (28.6)	59.4 (20.5)	64.6 (23.5)	83.8 (24.7)	88.1 (29.1)	70.7 (21.6)	45.9 (9.7)	50.1 (10.7)
**Yes**	70.8 (24.5)	76.7 (37.8)	67.5 (29.4)	56.4 (20.6)	62.5 (24.4)	81.5 (26.2)	85.6 (32.0)	68.3 (22.8)	44.7 (10.0)	49.2 (11.8)
**Prevalent comorbidity**										
**Coronary heart disease**										
**No**	73.4 (23.6)	78.8 (37.1)	71.3 (28.6)	59.3 (20.4)	64.4 (23.6)	83.5 (24.8)	87.7 (29.6)	70.4 (21.9)	45.8 (9.7)	49.9 (10.9)
**Yes**	60.0 (26.8)	69.3 (41.6)	62.5 (31.8)	44.7 (18.4)	57.2 (25.5)	77.6 (29.1)	81.8 (35.5)	64.2 (22.9)	40.6 (10.3)	48.3 (12.2)
**Stroke**										
**No**	73.0 (23.8)	78.7 (37.0)	71.1 (28.7)	58.8 (20.5)	64.3 (23.5)	83.5 (24.8)	87.6 (29.6)	70.2 (21.8)	45.7 (9.7)	49.9 (10.8)
**Yes**	58.8 (29.2)	60.4 (46.9)	58.6 (35.0)	45.5 (19.5)	52.0 (28.8)	68.8 (34.2)	77.5 (40.1)	62.0 (26.5)	39.5 (12.3)	46.1 (15.9)
**Diabetes mellitus**										
**No**	73.2 (23.8)	79.1 (36.9)	71.4 (28.8)	59.4 (20.4)	65.0 (23.4)	83.7 (24.7)	87.7 (29.5)	70.5 (22.0)	45.8 (9.7)	50.0 (10.9)
**Yes**	69.4 (24.7)	73.6 (39.8)	67.6 (29.1)	53.4 (20.9)	58.3 (25.0)	80.0 (27.2)	85.5 (32.5)	67.4 (22.0)	43.8 (10.4)	48.6 (11.5)
**Hip fracture**										
**No**	72.9 (23.9)	78.5 (37.1)	71.0 (28.8)	58.7 (20.5)	64.2 (23.8)	83.2 (25.1)	87.5 (29.8)	70.1 (22.0)	45.6 (9.7)	49.8 (11.0)
**Yes**	58.7 (23.9)	64.9 (45.4)	60.8 (32.7)	51.3 (22.6)	54.8 (18.0)	79.0 (28.1)	81.8 (36.8)	66.1 (21.5)	40.4 (11.2)	48.9 (12.1)
**Cancer**										
**No**	72.7 (24.1)	78.3 (37.3)	70.8 (28.9)	58.6 (20.6)	64.1 (23.7)	83.2 (25.1)	87.4 (30.0)	70.1 (22.0)	45.5 (9.8)	49.8 (11.0)
**Yes**	70.4 (20.5)	74.8 (40.2)	72.2 (27.4)	52.1 (20.7)	59.9 (25.2)	78.9 (26.6)	89.7 (26.9)	64.6 (19.9)	44.5 (9.6)	48.3 (9.2)
**Chronic obstructive pulmonary disease**										
**No**	73.9 (23.5)	80.0 (36.2)	71.9 (28.7)	59.8 (20.4)	65.3 (23.5)	83.9 (24.8)	87.9 (29.5)	70.6 (22.0)	46.1 (9.6)	50.0 (11.0)
**Yes**	64.4 (25.4)	66.9 (42.6)	64.2 (29.5)	50.3 (20.0)	55.7 (23.9)	78.3 (26.6)	84.4 (32.5)	66.3 (21.4)	41.6 (10.3)	48.7 (11.1)
**Osteoarthritis**										
**No**	78.9 (21.5)	84.9 (32.9)	81.4 (24.8)	63.3 (20.2)	69.5 (22.7)	87.5 (22.0)	91.3 (25.5)	74.2 (21.1)	48.5 (8.6)	51.1 (9.9)
**Yes**	67.7 (24.7)	73.0 (39.9)	62.4 (29.1)	54.7 (20.1)	59.7 (23.7)	79.6 (26.8)	84.3 (32.7)	66.8 (22.2)	43.2 (10.0)	48.8 (11.7)

^a^Abdominal obesity: waist circumference >102 cm in men and >88 cm in women.

### Short-term follow-up (2000–2001 to 2003)

After 2.5 years of follow-up, those who were never smokers or quit at least 15 years ago, and those with a healthy diet did not exhibit a positive association with HRQL. However, being very/moderately physically active was associated with better scores in all SF-36 scales and summaries. As regards non-traditional HB, sleeping 7–8 h/d was associated with better scores on physical role, social functioning, emotional role and the MCS. Also, people who spent seated <8 h/d scored better on bodily pain, and social functioning. Finally, those who saw their friends or neighbors daily scored worse on most SF-scales. Being physically active was the individual HB most strongly associated with better physical and mental HRQL (Table 2).

**Table 2:** Beta regression coefficients (95% confidence interval) of the SF-36 scales and summaries in 2003 according to traditional and non-traditional health behaviors in 2001 among older adults. (n = 2093).

	N/%	Physical functioning	Physical role	Bodily pain	General health	Vitality
**Traditional behavior**						
**Never smoking or quitting tobacco >15 y**						
**No**	566/27.0	Ref.	Ref.	Ref.	Ref.	Ref.
**Yes**	1527/73.0	0.18 (-2.40 to 2.76)	-2.29 (-6.61 to 2.04)	-3.08 (-6.25 to 0.09)	-0.11 (-2.27 to 2.05)	-0.82 (-3.46 to 1.82)
**Very/moderately active**						
**No**	401/19.2	Ref.	Ref.	Ref.	Ref.	Ref.
**Yes**	1692/80.8	6.50^c^ (3.92 to 9.17)	6.60^b^ (2.25 to 10.95)	3.60^a^ (0.42 to 6.77)	3.91^b^ (1.71 to 6.12)	6.54^c^ (3.85 to 9.22)
**Healthy diet score ≥ median in the cohort**						
**No**	1028/49.1	Ref.	Ref.	Ref.	Ref.	Ref.
**Yes**	1065/50.9	-0.12 (-1.98 to 1.75)	-1.72 (-4.84 to 1.40)	-0.98 (-3.27 to 1.31)	-0.62 (-2.17 to 0.94)	-0.14 (-2.05 to 1.77)
**Non-traditional behavior**						
**Sleeping 7–8 h/d**						
**No**	1195/57.1	Ref.	Ref.	Ref.	Ref.	Ref.
**Yes**	898/42.9	1.88 (-0.01 to 3.78)	4.30^b^ (1.13 to 7.48)	1.85 (-0.47 to 4.18)	0.52 (-1.06 to 2.11)	0.49 (-1.45 to 2.43)
**Sitting time <8h/d**						
**No**	208/10.0	Ref.	Ref.	Ref.	Ref.	Ref.
**Yes**	1884/90.0	2.48 (-0.81 to 5.76)	5.18 (-0.29 to 10.65)	4.70^a^ (0.68 to 8.73)	0.06 (-2.67 to 2.79)	1.00 (-2.35 to 4.35)
**Interaction with friends daily**						
**No**	313/15.0	Ref.	Ref.	Ref.	Ref.	Ref.
**Yes**	1780/85.0	-2.01 (-4.67 to 0.65)	-6.36^b^ (-10.81 to -1.91)	-1.78 (-5.04 to 1.49)	-2.77^a^ (-4.99 to– 0.55)	-4.71^b^ (-7.43 to -1.98)
**Traditional behaviors**						
**Never smoking or quitting tobacco >15 y**						
**No**	566/27.0	Ref.	Ref.	Ref.	Ref.	Ref.
**Yes**	1527/73.0	-2.10 (-5.11 to 0.92)	-0.03 (-4.29 to 4.23)	-1.93 (-4.35 to 0.49)	-0.16 (-1.01 to 0.68)	-0.73 (-1.99 to 0.52)
**Very/moderately active**						
**No**	401/19.2	Ref.	Ref.	Ref.	Ref.	Ref.
**Yes**	1692/80.8	4.64^b^ (1.59 to 7.70)	11.10^c^ (6.86 to 15.34)	3.59^b^ (1.16 to 6.03)	1.92^c^ (1.06 to 2.78)	2.61^c^ (1.35 to 3.86)
**Healthy diet score ≥ median in the cohort**						
**No**	1028/49.1	Ref.	Ref.	Ref.	Ref.	Ref.
**Yes**	898/42.9	0.20 (-1.97 to 2.38)	-0.54 (-3.62 to 2.53)	0.52 (-1.23 to 2.26)	-0.22 (-0.83 to 0.39)	0.09 (-0.82 to 1.00)
**Non-traditional behaviors**						
**Sleeping 7–8 h/d**						
**No**	1195/57.1	Ref.	Ref.	Ref.	Ref.	Ref.
**Yes**	898/42.9	3.07^b^ (0.86 to 5.28)	5.14^b^ (2.02 to 8.26)	1.29 (-0.49 to 3.06)	0.53 (-0.09 to 1.15)	1.21^a^ (0.29 to 2.13)
**Sitting time <8h/d**						
**No**	208/10.0	Ref.	Ref.	Ref.	Ref.	Ref.
**Yes**	1884/90.0	4.92^a^ (1.08 to 8.75)	2.04 (-3.35 to 7.43)	1.94 (-1.12 to 5.00)	1.00 (-0.07 to 2.08)	0.76 (-0.82 to 2.35)
**Daily interaction with friends**						
**No**	313/15.0	Ref.	Ref.	Ref.	Ref.	Ref.
**Yes**	1780/85.0	-2.27 (-5.37 to 0.84)	-5.22^a^ (-9.60 to -0.83)	-2.52^a^ (-5.02 to -0.03)	-0.90^a^ (-1.77to -0.03)	-1.75^b^ (-3.05 to -0.46)

^a^ p <0.05;

^b^ p <0.01;

^c^ p <0.001.

Models adjusted for age (years), sex, educational level (no formal education, primary education, secondary education or higher), occupational status (employed, unemployed, retired, housewife), alcohol intake (non drinker, ex-drinker, moderate consumption, excessive consumption), extreme sleep durations (no, yes), body mass index (<30 Kg/m^2^, ≥30 Kg/m^2^), waist circumference (no abdominal obesity, abdominal obesity), systolic blood pressure (<140 mmHg, ≥140 mmHg), hypercholesterolemia (no, yes), coronary heart disease (no, yes), stroke (no, yes), diabetes mellitus (no, yes), hip fracture (no, yes), cancer (no, yes), chronic obstructive pulmonary disease (no, yes), osteoarthritis (no, yes) and the corresponding scale of HRQL in 2001.

We decided to model the combined impact of those HB with a positive association with HRQL (being active, sleeping 7–8 h/d, and being seated <8 h/d). We found a strong and graded direct relationship between the number of HB and the score on most SF-36 scales (Table 3). Moreover, compared with individuals with ≤1 HB, the β (95% confidence interval) for the score on the PCS of the SF-36 was 1.42 (0.52 to 2.33) in those with 2 HB, and 2.06 (1.09 to 3.03) in those with 3 HB, p-trend <0.001. Corresponding figures for the MCS were 1.89 (0.58 to 3.21) for 2 HB, and 3.35 (1.95 to 4.76) for 3 HB, p-trend <0.001 (Table 3). To place these results into context, we compared the magnitude of this association with the expected decline in HRQL over time (as age increases). For instance, in our cohort, the average decrease in the physical functioning scale was 0.71 points for the increase in one year in the age of participants; thus, in Table 3 the β coefficient for 3 positive HB of 7.18 corresponds to a 10-year improvement in physical functioning.

**Table 3:** Beta regression coefficients (95% confidence interval) of the SF-36 scales and summaries in 2003 according to the number of positive health behaviours in 2001 among older adults. (n = 2093).

Number of positive healthy behaviors	N/%	Physical functioning	Physical role	Bodily pain	General health	Vitality
**0–1**	352/16.8	Ref.	Ref.	Ref.	Ref.	Ref.
**2**	1016/48.5	5.01^c^ (2.24 to 7.77)	6.10^b^ (1.53 to 10.66)	3.24 (-0.11 to 6.58)	1.80 (-0.49 to 4.09)	4.58^b^ (1.76 to 7.40)
**3**	725/34.6	7.18^c^ (4.21 to 10.15)	10.5^c^ (5.62 to 15.38)	5.29 ^b^ (1.73 to 8.84)	2.76^a^ (0.29 to 5.23)	5.14^b^ (2.10 to 8.18)
**P for trend**		<0.001	<0.001	0.004	0.033	0.004
**Number of positive healthy behaviors**	**N/%**	**Social functioning**	**Emotional role**	**Mental health**	**Physical summary**	**Mental summary**
**0–1**	352/16.8	Ref.	Ref.	Ref.	Ref.	Ref.
**2**	1016/48.5	5.30^b^ (2.07 to 8.52)	7.98^c^ (3.53 to 12.44)	2.53^a^ (-0.00 to 5.07)	1.42^b^ (0.52 to 2.33)	1.89^b^ (0.58 to 3.21)
**3**	725/34.6	8.39^c^ (4.96 to 11.82)	14.36^c^ (9.62 to 19.10)	4.26^b^ (1.54 to 6.97)	2.06^c^ (1.09 to 3.03)	3.35^c^ (1.95 to 4.76)
**P for trend**		<0.001	<0.001	0.002	<0.001	<0.001

^a^ p <0.05;

^b^ p <0.01;

^c^ p <0.001.

Models adjusted for age (years), sex, educational level (no formal education, primary education, secondary education or higher), occupational status (employed, unemployed, retired, housewife), alcohol intake (non drinker, ex-drinker, moderate consumption, excessive consumption), extreme sleep durations (no, yes), body mass index (<30 Kg/m^2^, ≥30 Kg/m^2^), waist circumference (no abdominal obesity, abdominal obesity), systolic blood pressure (<140 mmHg, ≥140 mmHg), hypercholesterolemia (no, yes), coronary heart disease (no, yes), stroke (no, yes), diabetes mellitus (no, yes), hip fracture (no, yes), cancer (no, yes), chronic obstructive pulmonary disease (no, yes), osteoarthritis (no, yes) and the corresponding scale of HRQL in 2001.

^d^ Being very/moderate physically active, sleeping 7–8 hours, and sitting time <8h/d were considered as healthy behaviours.

### Long-term follow-up (2000–2001 to 2009)

After an 8.5-year follow-up, never being a smoker or quitting tobacco >15 year ago was associated with worse score in the emotional role scale and in MCS. A healthy diet, adequate sleep duration, short time spent seated, and frequently seeing friends and neighbors did not show an association with HRQL. However, being very/moderately physically active was linked to better scores on most physical dimensions of the SF-36: physical functioning, physical role, bodily pain, general health, vitality, social function, emotional role, and the PCS (Table 4). Given that the average decrease in the physical functioning score from 2000–2001 to 2009 was 1.06 points per year of follow-up, the β coefficient associated with being very/moderately physically active (β = 10.65) equals to a 10-year improvement in this scale.

**Table 4:** Beta regression coefficients (95% confidence interval) of the SF-36 scales and summaries in 2009 according to traditional and non-traditional healthy behaviours in 2001 among older adults. (n = 993).

	N/%	Physical functioning	Physical role	Bodily pain	General health	Vitality
**Traditional behavior**						
**Never smoking or quitting tobacco >15 y**						
**No**	248/25.0	Ref.	Ref.	Ref.	Ref.	Ref.
**Yes**	745/75.0	2.54 (-1.81 to 6.90)	-2.00 (-9.49 to 5.49)	0.33 (-4.81 to 5.48)	-1.22 (-4.20 to 1.77)	-1.06 (-5.40 to 3.27)
**Very/moderately active**						
**No**	132/13.3	Ref.	Ref.	Ref.	Ref.	Ref.
**Yes**	861/86.7	10.65^b^ (5.87 to 15.44)	15.30^b^ (7.09 to 23.51)	6.31^a^ (0.72 to 11.90)	7.14^b^ (3.82 to 10.45)	9.88^b^ (5.12 to 14.65)
**Healthy diet score ≥ median in the cohort**						
**No**	447/45.0	Ref.	Ref.	Ref.	Ref.	Ref.
**Yes**	547/55.0	0.22 (-2.86 to 3.31)	0.14 (-5.18 to 5.46)	-3.10 (-6.75 to 0.55)	-0.74 (-2.85 to 1.37)	-0.96 (-4.02 to 2.10)
**Non-traditional behavior**						
**Sleeping 7–8 h/d**						
**No**	554/55.8	Ref.	Ref.	Ref.	Ref.	Ref.
**Yes**	439/44.2	1.54 (-1.57 to 4.65)	0.03 (-5.33 to 5.38)	0.89 (-2.78 to 4.56)	0.32 (-1.81 to 2.46)	1.77 (-1.33 to 4.87)
**Sitting time <8h/d**						
**No**	63/6.4	Ref.	Ref.	Ref.	Ref.	Ref.
**Yes**	930/93.6	0.84 (-5.59 to 7.28)	8.22 (-2.87 to 19.31)	-1.55 (-9.16 to 6.06)	0.90 (-3.50 to 5.31)	-3.55 (-9.95 to 2.86)
**Interaction with friends daily**						
**No**	134/13.5	Ref.	Ref.	Ref.	Ref.	Ref.
**Yes**	859/86.5	-3.36 (-7.97 to 1.25)	-5.23 (-13.15 to 2.70)	0.87 (-4.57 to 6.32)	-1.88 (-5.04 to 1.27)	-1.32 (-5.91 to 3.28)
**Traditional behavior**						
**Never smoking or quitting tobacco >15 y**						
**No**	248/25.0	Ref.	Ref.	Ref.	Ref.	Ref.
**Yes**	745/75.0	-0.13 (-4.92 to 4.67)	-6.47^a^ (-12.69 to -0.24)	-2.76 (-6.74 to 1.21)	0.98 (-0.82 to 2.78)	-2.18^a^ (-4.27 to -0.08)
**Very/moderately active**						
**No**	132/13.3	Ref.	Ref.	Ref.	Ref.	Ref.
**Yes**	861/86.7	5.63^a^ (0.36 to 10.90)	7.88^a^ (1.10 to 14.66)	1.79 (-2.55 to 6.14)	4.24^b^ (2.27 to 6.22)	1.20 (-1.08 to 3.49)
**Healthy diet score ≥ median in the cohort**						
**No**	447/45.0	Ref.	Ref.	Ref.	Ref.	Ref.
**Yes**	547/55.0	-0.68 (-4.07 to 2.71)	-0.98 (-5.39 to 3.42)	-1.22 (-4.04 to 1.60)	-0.03 (-1.31 to 1.25)	-0.54 (-2.03 to 0.94)
**Non-traditional behavior**						
**Sleeping 7–8 h/d**						
**No**	554/55.8	Ref.	Ref.	Ref.	Ref.	Ref.
**Yes**	439/44.2	1.77 (-1.65 to 5.19)	-0.39 (-4.83 to 4.05)	1.14 (-1.70 to 3.98)	0.30 (-0.99 to 1.58)	0.45 (-1.05 to 1.94)
**Sitting time <8h/d**						
**No**	63/6.4	Ref.	Ref.	Ref.	Ref.	Ref.
**Yes**	930/93.6	1.00 (-6.10 to 8.09)	-3.98 (-13.20 to 5.23)	0.79 (-5.09 to 6.66)	1.01 (-1.65 to 3.67)	-1.32 (-4.41 to 1.77)
**Interaction with friends daily**						
**No**	134/13.5	Ref.	Ref.	Ref.	Ref.	Ref.
**Yes**	859/86.5	-1.61 (-6.69 to 3.47)	4.33 (-2.26 to 10.93)	-0.52 (-4.73 to 3.69)	-1.51 (-3.41 to 0.39)	0.79 (-1.43 to 3.01)

^a^ p <0.05;

^b^ p <0.001.

Models adjusted for age (years), sex, educational level (no formal education, primary education, secondary education or higher), occupational status (employed, unemployed, retired, housewife), alcohol intake (non drinker, ex-drinker, moderate consumption, excessive consumption), extreme sleep durations (no, yes), body mass index (<30 Kg/m^2^, ≥30 Kg/m^2^), waist circumference (no abdominal obesity, abdominal obesity), systolic blood pressure (<140 mmHg, ≥140 mmHg), hypercholesterolemia (no, yes), coronary heart disease (no, yes), stroke (no, yes), diabetes mellitus (no, yes), hip fracture (no, yes), cancer (no, yes), chronic obstructive pulmonary disease (no, yes), osteoarthritis (no, yes) and the corresponding scale of HRQL in 2001.

## Discussion

Among older adults in Spain, a greater number of traditional and non-traditional HB (being active, adequate sleep duration, and being non-sedentary) were linked to better short-term HRQL. Also, being physically active showed a long-term association with better HRQL. However, several HB that in previous studies were associated with lower mortality, such as non-smoking, a healthy diet, or frequent contact with friends and neighbors, did not show a relation to HRQL.

### Traditional health behaviors

In several cohort studies in older adults, smoking has been associated with higher mortality and morbidity, and lower HRQL.[32–35] However, in our study having never being a smoker or former smoker showed a worse score in bodily pain, although this association did not reached statistical significance. This observation might be due to survival bias, whereby some smokers die early in life and those who reach old age represent a subset of individuals who are partly resistant to the effects of tobacco.[34] Also, some former smokers might have quit because of health reasons, which may have reduced HRQL.[36] In fact, while smokers in the Nurses’ cohort study had lower HRQL, those who quit did not improve HRQL during a 21-year follow-up.[37] Moreover, non-smokers might suffer greater musculoskeletal pain because of a higher body weight than smokers. Notwithstanding this, the association between smoking and pain is still unclear because in experimental research nicotine has shown some analgesic effects, but in epidemiological studies smoking has been linked to chronic pain.[38]

Only three studies have assessed prospectively the association between a healthy diet and HRQL.[39–41] The first one is a small prospective analysis nested in the DASH (Diet to Stop Hypertension) trial. Individuals following a "combination diet", which emphasized fruit, vegetables, and low-fat dairy products, improved their HRQL modestly compared to those following the typical American diet. However, the mean age of participants in this analysis was only 45.[39] In the second study, that included younger subjects (mean age 40), a Mediterranean diet was also linked to a slightly better HRQL.[40] And in the third study, conducted among individuals with a mean age of 67, a high adherence to the Australian Guide to Healthy Eating was associated with better physical health.[41] However, in the latter two studies, HRQL was not assessed at baseline, so the observed results might be explained by baseline differences in HRQL across dietary groups. Finally, in our cohort, a healthy diet was not associated with better HRQL. It is possible that those with a higher adherence to a healthy diet did so because of health reasons. So that the well-known benefits of a healthy diet in reducing morbidity could be compensated for the impact on HRQL of health disorders that led to a better diet. Indeed, in our cohort, the number of chronic conditions suffered by those with a healthy diet was slightly greater than in those without one. To address this issue, the analyses were adjusted for self-reported morbidity and HRQL at baseline. Unfortunately, we did not collect data on the severity and duration of diseases or on dietary changes before study initiation.

Like in our cohort, other studies in older adults have shown that regular PA is associated with better HRQL;[42]and there is also evidence of a direct dose-response between PA and HRQL, particularly on physical functioning and vitality, which is observed even if walking is the only activity performed.[43] Moreover, accelerometry-based light PA has been associated with better self-reported health after controlling for moderate-vigorous PA and sedentary behavior. [44] Regarding mental well-being, a meta-analysis of the literature has shown that PA improves mental well-being even in the frail elderly.[45]

### Non-traditional healthy behaviors

In a systematic review of the literature, sleep duration has shown a U-shaped relationship with mortality.[17] Also in several cross-sectional studies both short and long sleep durations were related to poor self-rated health and lower HRQL.[46,47] However, very few studies have examined this association prospectively. In our cohort, we had already reported that extreme sleep durations (≤ 5 or ≥ 10 hours) are linked to worse HRQL in the elderly.[48] Moreover, in another longitudinal study, short sleep (< 7 h/d) as well as long sleep (> 8 h/d) sleep were associated with worse late life cognitive function.[49] In line with these findings, our results show that sleeping 7–8 h/d is associated with better scores on most scales of HRQL in the short-term. However, the magnitude of this association decreased in the long-term, perhaps because sleep needs progressively decline with age (in 2009 the mean age of participants was 80).

Only a few studies have examined the association between a sedentary lifestyle and HRQL. In a cross-sectional study with 3,796 Australian subjects aged ≥ 18, PA had a stronger association with HRQL than screen-time, but HRQL was worse in those who simultaneously did no PA and had a high screen-time.[50] Also, a previous analysis in our cohort showed that, after controlling for PA, the number of sitting hours had a gradual and inverse relationship with physical functioning, physical role, bodily pain, vitality, social functioning, and mental health.[12] Lastly, even among adults who do the recommended PA, those with prolonged sitting time gain greater weight, which in turn could reduce HRQL.[51] Thus, both increasing PA and decreasing sitting time might improve HRQL.

A poor social network has been associated with increased risk of death.[52] In a previous cross-sectional analysis in this cohort, HRQL was lower among those who seldom or never saw friends or neighbors,[53] but this association is no longer held in this prospective analysis. In fact, in old age frequent interaction with friends or neighbors could simply be a marker of poor health; in this case, it could indicate previous poor physical condition that remained during the follow-up.[54]

### Combined HB and HRQL

We are aware of only two previous studies that evaluated the joint impact of several HB on HRQL, and they were conducted among middle-age individuals. The "Midlife in the United States" study (MIDUS) using the SF-36 assessed the joint contribution of control beliefs, social support and physical exercise on physical functioning. Individuals in better categories in the studied variables experienced a smaller decline in physical function after 10 years of follow-up, either when these variables were considered individually or as an aggregate.[9] The second study was a cross-sectional analysis of data on some traditional and non-traditional HB (smoking, physical activity, diet, sitting time, sleep duration and sleep quality) among 10,478 individuals; those with a greater number of unhealthy behaviors had a higher prevalence of poor self-rated health, and higher frequency of "unhealthy" days. When these behaviors were considered individually, bad sleep quality and low PA had the strongest associations. However, reverse causation cannot be ruled out due to the cross-sectional design.[10]

### Strengths and limitations

Strengths of this study were the prospective design, data collection with validated and standardized methods, and that the analyses were adjusted for many potential confounders, including baseline HRQL. This study also had some limitations. First, as in other studies with older adults, the size of the cohort decreased over time because of a substantial number of deaths and losses to follow-up. Second, we assumed that HB were stable over time. However, given that some HB deteriorate over time (e.g., PA decreases during follow-up), the association between HB and HRQL has probably been underestimated. And third, the data were self-reported, that might be affected by recall bias.

### Practical implications

While the aggregation of traditional and non-traditional HB seems to be more important to prevent mortality, HB related to movement and rest could help in promoting HRQL among older persons. PA seems to be the pivotal HB to improve HRQL among older adults.

## Supporting Information

S1 Dataset One dateset is available. (DTA)
