# Correction: Combined Impact of Traditional and Non-Traditional Healthy Behaviors on Health-Related Quality of Life: A Prospective Study in Older Adults

**DOI:** 10.1371/journal.pone.0173850

**Published:** 2017-03-08

**Authors:** Ana Bayán-Bravo, Raúl F. Pérez-Tasigchana, Carmen Sayón-Orea, David Martínez-Gómez, Esther López-García, Fernando Rodríguez-Artalejo, Pilar Guallar-Castillón

There is an error in Table 2. The second header row is missing. Please see the corrected Table 2 here.

**Table 2:** Beta regression coefficients (95% confidence interval) of the SF-36 scales and summaries in 2003 according to traditional and non-traditional health behaviors in 2001 among older adults. (n = 2093).

	**N/%**	**Physical functioning**	**Physical role**	**Bodily pain**	**General health**	**Vitality**
**Traditional behavior**						
**Never smoking or quitting tobacco >15 y**						
**No**	566/27.0	Ref.	Ref.	Ref.	Ref.	Ref.
**Yes**	1527/73.0	0.18 (-2.40 to 2.76)	-2.29 (-6.61 to 2.04)	-3.08 (-6.25 to 0.09)	-0.11 (-2.27 to 2.05)	-0.82 (-3.46 to 1.82)
**Very/moderately active**						
**No**	401/19.2	Ref.	Ref.	Ref.	Ref.	Ref.
**Yes**	1692/80.8	6.50^c^ (3.92 to 9.17)	6.60^b^ (2.25 to 10.95)	3.60^a^ (0.42 to 6.77)	3.91^b^ (1.71 to 6.12)	6.54^c^ (3.85 to 9.22)
**Healthy diet score ≥ median in the cohort**						
**No**	1028/49.1	Ref.	Ref.	Ref.	Ref.	Ref.
**Yes**	1065/50.9	-0.12 (-1.98 to 1.75)	-1.72 (-4.84 to 1.40)	-0.98 (-3.27 to 1.31)	-0.62 (-2.17 to 0.94)	-0.14 (-2.05 to 1.77)
**Non-traditional behavior**						
**Sleeping 7–8 h/d**						
**No**	1195/57.1	Ref.	Ref.	Ref.	Ref.	Ref.
**Yes**	898/42.9	1.88 (-0.01 to 3.78)	4.30^b^ (1.13 to 7.48)	1.85 (-0.47 to 4.18)	0.52 (-1.06 to 2.11)	0.49 (-1.45 to 2.43)
**Sitting time <8h/d**						
**No**	208/10.0	Ref.	Ref.	Ref.	Ref.	Ref.
**Yes**	1884/90.0	2.48 (-0.81 to 5.76)	5.18 (-0.29 to 10.65)	4.70^a^ (0.68 to 8.73)	0.06 (-2.67 to 2.79)	1.00 (-2.35 to 4.35)
**Interaction with friends daily**						
**No**	313/15.0	Ref.	Ref.	Ref.	Ref.	Ref.
**Yes**	1780/85.0	-2.01 (-4.67 to 0.65)	-6.36^b^ (-10.81 to -1.91)	-1.78 (-5.04 to 1.49)	-2.77^a^ (-4.99 to– 0.55)	-4.71^b^ (-7.43 to -1.98)
	**N/%**	**Social functioning**	**Emotional role**	**Mental health**	**Physical Summary**	**Mental Summary**
**Traditional behaviors**						
**Never smoking or quitting tobacco >15 y**						
**No**	566/27.0	Ref.	Ref.	Ref.	Ref.	Ref.
**Yes**	1527/73.0	-2.10 (-5.11 to 0.92)	-0.03 (-4.29 to 4.23)	-1.93 (-4.35 to 0.49)	-0.16 (-1.01 to 0.68)	-0.73 (-1.99 to 0.52)
**Very/moderately active**						
**No**	401/19.2	Ref.	Ref.	Ref.	Ref.	Ref.
**Yes**	1692/80.8	4.64^b^ (1.59 to 7.70)	11.10‡ (6.86 to 15.34)	3.59^b^ (1.16 to 6.03)	1.92‡ (1.06 to 2.78)	2.61^c^ (1.35 to 3.86)
**Healthy diet score ≥ median in the cohort**						
**No**	1028/49.1	Ref.	Ref.	Ref.	Ref.	Ref.
**Yes**	898/42.9	0.20 (-1.97 to 2.38)	-0.54 (-3.62 to 2.53)	0.52 (-1.23 to 2.26)	-0.22 (-0.83 to 0.39)	0.09 (-0.82 to 1.00)
**Non-traditional behaviors**						
**Sleeping 7–8 h/d**						
**No**	1195/57.1	Ref.	Ref.	Ref.	Ref.	Ref.
**Yes**	898/42.9	3.07^b^ (0.86 to 5.28)	5.14^b^ (2.02 to 8.26)	1.29 (-0.49 to 3.06)	0.53 (-0.09 to 1.15)	1.21^a^ (0.29 to 2.13)
**Sitting time <8h/d**						
**No**	208/10.0	Ref.	Ref.	Ref.	Ref.	Ref.
**Yes**	1884/90.0	4.92^a^ (1.08 to 8.75)	2.04 (-3.35 to 7.43)	1.94 (-1.12 to 5.00)	1.00 (-0.07 to 2.08)	0.76 (-0.82 to 2.35)
**Daily interaction with friends**						
**No**	313/15.0	Ref.	Ref.	Ref.	Ref.	Ref.
**Yes**	1780/85.0	-2.27 (-5.37 to 0.84)	-5.22^a^ (-9.60 to -0.83)	-2.52^a^ (-5.02 to -0.03)	-0.90^a^ (-1.77to -0.03)	-1.75^b^ (-3.05 to -0.46)

^a^ p <0.05;

^b^ p <0.01;

^c^ p <0.001.

Models adjusted for age (years), sex, educational level (no formal education, primary education, secondary education or higher), occupational status (employed, unemployed, retired, housewife), alcohol intake (non drinker, ex-drinker, moderate consumption, excessive consumption), extreme sleep durations (no, yes), body mass index (<30 Kg/m^2^, ≥30 Kg/m^2^), waist circumference (no abdominal obesity, abdominal obesity), systolic blood pressure (<140 mmHg, ≥140 mmHg), hypercholesterolemia (no, yes), coronary heart disease (no, yes), stroke (no, yes), diabetes mellitus (no, yes), hip fracture (no, yes), cancer (no, yes), chronic obstructive pulmonary disease (no, yes), osteoarthritis (no, yes) and the corresponding scale of HRQL in 2001.

There is an error in Table 4. The second header row is missing. Please see the corrected Table 4 here.

**Table 4:** Beta regression coefficients (95% confidence interval) of the SF-36 scales and summaries in 2009 according to traditional and non-traditional healthy behaviours in 2001 among older adults. (n = 993).

	**N/%**	**Physical functioning**	**Physical role**	**Bodily pain**	**General health**	**Vitality**
**Traditional behavior**						
**Never smoking or quitting tobacco >15 y**						
**No**	248/25.0	Ref.	Ref.	Ref.	Ref.	Ref.
**Yes**	745/75.0	2.54 (-1.81 to 6.90)	-2.00 (-9.49 to 5.49)	0.33 (-4.81 to 5.48)	-1.22 (-4.20 to 1.77)	-1.06 (-5.40 to 3.27)
**Very/moderately active**						
**No**	132/13.3	Ref.	Ref.	Ref.	Ref.	Ref.
**Yes**	861/86.7	10.65^b^ (5.87 to 15.44)	15.30^b^ (7.09 to 23.51)	6.31^a^ (0.72 to 11.90)	7.14^b^ (3.82 to 10.45)	9.88^b^ (5.12 to 14.65)
**Healthy diet score ≥ median in the cohort**						
**No**	447/45.0	Ref.	Ref.	Ref.	Ref.	Ref.
**Yes**	547/55.0	0.22 (-2.86 to 3.31)	0.14 (-5.18 to 5.46)	-3.10 (-6.75 to 0.55)	-0.74 (-2.85 to 1.37)	-0.96 (-4.02 to 2.10)
**Non-traditional behavior**						
**Sleeping 7–8 h/d**						
**No**	554/55.8	Ref.	Ref.	Ref.	Ref.	Ref.
**Yes**	439/44.2	1.54 (-1.57 to 4.65)	0.03 (-5.33 to 5.38)	0.89 (-2.78 to 4.56)	0.32 (-1.81 to 2.46)	1.77 (-1.33 to 4.87)
**Sitting time <8h/d**						
**No**	63/6.4	Ref.	Ref.	Ref.	Ref.	Ref.
**Yes**	930/93.6	0.84 (-5.59 to 7.28)	8.22 (-2.87 to 19.31)	-1.55 (-9.16 to 6.06)	0.90 (-3.50 to 5.31)	-3.55 (-9.95 to 2.86)
**Interaction with friends daily**						
**No**	134/13.5	Ref.	Ref.	Ref.	Ref.	Ref.
**Yes**	859/86.5	-3.36 (-7.97 to 1.25)	-5.23 (-13.15 to 2.70)	0.87 (-4.57 to 6.32)	-1.88 (-5.04 to 1.27)	-1.32 (-5.91 to 3.28)
	**N / %**	**Social functioning**	**Emotional role**	**Mental health**	**Physical Summary**	**Mental Summary**
**Traditional behavior**						
**Never smoking or quitting tobacco >15 y**						
** No**	248/25.0	Ref.	Ref.	Ref.	Ref.	Ref.
** Yes**	745/75.0	-0.13 (-4.92 to 4.67)	-6.47^a^ (-12.69 to -0.24)	-2.76 (-6.74 to 1.21)	0.98 (-0.82 to 2.78)	-2.18^a^ (-4.27 to -0.08)
**Very/moderately active**						
** No**	132/13.3	Ref.	Ref.	Ref.	Ref.	Ref.
** Yes**	861/86.7	5.63^a^ (0.36 to 10.90)	7.88^a^ (1.10 to 14.66)	1.79 (-2.55 to 6.14)	4.24^b^ (2.27 to 6.22)	1.20 (-1.08 to 3.49)
**Healthy diet score ≥ median in the cohort**						
** No**	447/45.0	Ref.	Ref.	Ref.	Ref.	Ref.
** Yes**	547/55.0	-0.68 (-4.07 to 2.71)	-0.98 (-5.39 to 3.42)	-1.22 (-4.04 to 1.60)	-0.03 (-1.31 to 1.25)	-0.54 (-2.03 to 0.94)
**Non-traditional behavior**						
**Sleeping 7–8 h/d**						
** No**	554/55.8	Ref.	Ref.	Ref.	Ref.	Ref.
** Yes**	439/44.2	1.77 (-1.65 to 5.19)	-0.39 (-4.83 to 4.05)	1.14 (-1.70 to 3.98)	0.30 (-0.99 to 1.58)	0.45 (-1.05 to 1.94)
**Sitting time <8h/d**						
** No**	63/6.4	Ref.	Ref.	Ref.	Ref.	Ref.
** Yes**	930/93.6	1.00 (-6.10 to 8.09)	-3.98 (-13.20 to 5.23)	0.79 (-5.09 to 6.66)	1.01 (-1.65 to 3.67)	-1.32 (-4.41 to 1.77)
**Interaction with friends daily**						
** No**	134/13.5	Ref.	Ref.	Ref.	Ref.	Ref.
** Yes**	859/86.5	-1.61 (-6.69 to 3.47)	4.33 (-2.26 to 10.93)	-0.52 (-4.73 to 3.69)	-1.51 (-3.41 to 0.39)	0.79 (-1.43 to 3.01)

^a^ p <0.05;

^b^ p <0.001.

Models adjusted for age (years), sex, educational level (no formal education, primary education, secondary education or higher), occupational status (employed, unemployed, retired, housewife), alcohol intake (non drinker, ex-drinker, moderate consumption, excessive consumption), extreme sleep durations (no, yes), body mass index (<30 Kg/m^2^, ≥30 Kg/m^2^), waist circumference (no abdominal obesity, abdominal obesity), systolic blood pressure (<140 mmHg, ≥140 mmHg), hypercholesterolemia (no, yes), coronary heart disease (no, yes), stroke (no, yes), diabetes mellitus (no, yes), hip fracture (no, yes), cancer (no, yes), chronic obstructive pulmonary disease (no, yes), osteoarthritis (no, yes) and the corresponding scale of HRQL in 2001.

## References

[1] Bayán-Bravo A, Pérez-Tasigchana RF, Sayón-Orea C, Martínez-Gómez D, López-García E, Rodríguez-Artalejo F, et al (2017) Combined Impact of Traditional and Non-Traditional Healthy Behaviors on Health-Related Quality of Life: A Prospective Study in Older Adults. PLoS ONE 12(1): e0170513 doi: 10.1371/journal.pone.0170513 28122033 10.1371/journal.pone.0170513 PMC5266310

